# Rapid Shift from SARS-CoV-2 Delta to Omicron Sub-Variants within a Dynamic Southern U.S. Borderplex

**DOI:** 10.3390/v15030658

**Published:** 2023-02-28

**Authors:** Elisa Robles-Escajeda, Jonathon E. Mohl, Lisett Contreras, Ana P. Betancourt, Bibiana M. Mancera, Robert A. Kirken, Georgialina Rodriguez

**Affiliations:** 1 The Department of Biological Sciences, The University of Texas at El Paso, 500 W. University Ave, El Paso, TX 79968, USA; 2 Border Biomedical Research Center, The University of Texas at El Paso, 500 W. University Ave, El Paso, TX 79968, USA; 3 The Department of Mathematical Sciences, The University of Texas at El Paso, 500 W. University Ave, El Paso, TX 79968, USA

**Keywords:** SARS-CoV-2, COVID-19, Omicron, Delta, next-generation sequencing (NGS), genomic surveillance

## Abstract

COVID-19, caused by the Severe Acute Respiratory Syndrome Coronavirus 2 (SARS-CoV-2), remains an ongoing global health challenge. This study analyzed 3641 SARS-CoV-2 positive samples from the El Paso, Texas, community and hospitalized patients over 48 weeks from Fall 2021 to Summer 2022. The binational community along the U.S. southern border was predominantly SARS-CoV-2 Delta variant (B.1.617.2) positive for a 5-week period from September 2021 to January 2022 and quickly transitioned to the Omicron variant (B.1.1.529), which was first detected at the end of December 2021. Omicron replaced Delta as the predominant detectable variant in the community and was associated with a sharp increase in COVID-19 positivity rate, related hospitalizations, and newly reported cases. In this study, Omicron BA.1, BA.4, and BA.5 variants were overwhelmingly associated with S-gene dropout by qRT-PCR analysis unlike the Delta and Omicron BA.2 variants. The study reveals that a dominant variant, like Delta, can be rapidly replaced by a more transmissible variant, like Omicron, within a dynamic metropolitan border city, necessitating enhanced monitoring, readiness, and response from public health officials and healthcare workers.

## 1. Introduction

El Paso, Texas, resides along the southern United States–Mexico border. Its name is derived from El Paso del Norte, “Passage to the North”, and together with its neighbor city Ciudad Juarez, Chihuahua, Mexico, represents a major corridor connecting South America to Northern America and Eastern to Western United States (U.S.). This unique borderplex area is geographically separated by the Rio Grande River, a junction between two countries (U.S. and Mexico), two U.S. southern states (Texas and New Mexico), one northern Mexican state (Chihuahua), and three counties (El Paso, Doña Ana, and Juarez). The border community is home to nearly 2 million people (El Paso, Texas, at 867,947 and Ciudad Juarez, Chihuahua, Mexico, at 1,166,246, 2022 data). U.S. ports of entry in El Paso, TX, see more than approximately 35,000 passenger vehicles, 2600 commercial vehicles, and 19,000 pedestrians commuting daily for educational, work, social, and commerce purposes [1]. Close familial, cultural, and commercial ties between the neighboring communities tightly link the area through the six international bridges connecting El Paso and Juarez residents on both sides of the border. 

El Paso city’s first case of SARS-CoV-2 was reported on 13 March 2020. Within one week, both Ciudad Juarez, Mexico, and the neighboring county of Doña Ana, New Mexico, announced their first cases. The proximity of these events is unsurprising given the juxtaposition and economic dependence of the communities. This study sought to track the progression of SARS-CoV-2 variants employing Next-Generation Sequencing (NGS) surveillance. The Centers for Disease Control (CDC) reported SARS-CoV-2 variants using WHO labels and the Pango lineage naming system based on their ability to spread, severity of the disease, response to available treatments, and vaccine protection. Two new variants of concern (VOC) emerged within the U.S. in 2021. The Delta (Δ) variant (B.1.617.2 and AY lineages) became a VOC in June 2021 [2]. Subsequently, the more contagious Omicron (O) variant (B.1.1.529) became a VOC in November 2021 while spreading quickly and becoming the dominant U.S. variant [2,3]. The Delta and Omicron variant share four mutations in the spike protein (T95I, K417N, D614G, and P681R); however, there are 10 mutations unique to Delta and 29 mutations unique to Omicron [2,4]. Both Δ-B.1.617.2 and O-B.1.1.529 contain the aspartic acid (D) substitution to glycine (G) at position 614 in the viral spike protein (D614G). This missense mutation has been associated with enhanced replication, transmission [5,6,7], and infectivity of the virus [7,8]. Structural and functional protein analysis of the D614G mutation indicated that this mutation promotes conformational changes for binding to ACE2 and viral fusion with target cells [9]. Mutations in the ancestral viral sequence led to the classification of lineages and sublineages [10,11]. Lineages for the Delta variant included Δ-B.1.617.2 and various Δ-AY lineages. Major lineages of the Omicron variant included O-BA.1, O-BA.2, O-BA.3, O-BA.4, and O-BA.5 along with a multitude of sublineages. Circulating variants quickly shifted with the Delta variant cases to the Omicron variant in the U.S. The Delta variant was first detected in the U.S. in March 2021 and rapidly became the predominant variant, increasing the number of cases, hospitalizations, and deaths nationally. In November 2021, Omicron was first detected then became the major circulating variant during the week of 20 December 2021 in the U.S. [3,12]. In Texas, the earliest reports of Delta occurred during May 2021 [13], which was followed by Omicron, where the first case was reported on the first week of December 2021 [14]. The first report of the Delta variant in El Paso, Texas, occurred in July 2021 [15]. Finally, Omicron cases were initially reported in El Paso County from specimens collected on December 21 and 22, 2021 [16].

The aim of this study was to track the progression of new variants within the dynamic border community through genomic sequencing to inform city and county public health in support of management decisions. This intensive work conducted over a 48-week period (2021–2022) indicated that the dominant Delta variant was rapidly replaced by Omicron within El Paso County. During the second week (wk) of December 2021 (wk 49-2021), NGS sequenced samples were all the Delta variant (Δ-AY lineages). Subsequently, by 3 January 2022 (wk 01-2022), an average of 89% of samples sequenced (general population and inpatient) were identified as the Omicron variant (O-BA.1). This report serves as an example of how quickly any dominant viral variant can be replaced in a metropolitan, bi-national community. Undoubtedly, the emergence of rapidly transmissible SARS-CoV-2 variants requires a robust monitoring program of progression in dynamic regions, such as the southern U.S. border.

## 2. Materials and Methods

### 2.1. Sample Collection

The University of Texas at El Paso’s Border Biomedical Research Center (UTEP-BBRC) collaborated with the University Medical Center (UMC) and the El Paso Department of Public Health (DPH) to sequence SARS-CoV-2 for epidemiological surveillance within the El Paso, Texas, community under the guidance of UTEP’s Institutional Review Board approved protocol between September 2021 to July 2022. Specimens were included in the study if they were collected in the El Paso, TX, area from either the UMC hospital or DPH and identified as SARS-CoV-2 positive. All other specimens were excluded. UMC provided positive COVID-19 samples obtained from inpatient hospitalized individuals, whereas DPH provided positive COVID-19 samples from non-hospitalized individuals. All samples were de-identified of any personal and clinical information. Oropharyngeal and nasopharyngeal swabs were placed in viral transport media (VTM) or Bionex solutions for diagnostic processing by the provider. COVID-19 positive samples were provided to the UTEP-BBRC for viral RNA extraction followed by next generation sequencing (NGS) and analysis.

### 2.2. Viral RNA Extraction and SARS-CoV-2 Confirmation

To validate the positivity of the received samples, we performed quantitative real-time PCR (qRT-PCR). SARS-CoV-2 RNA was isolated from specimens (200 μL) utilizing the MagMAX Viral/Pathogen Nucleic Acid Isolation kit (ThermoFisher, Waltham, MA, USA) and the KingFisher Flex Purification System (ThermoFisher) according to the manufacturer’s instructions. Viral SARS-CoV-2 RNA was confirmed by multiplex quantitative RT-PCR test following the guidelines of the Applied Biosciences TaqPath COVID-19 Combo Kit approved for detection of SARS-CoV-2 under Emergency Use Authorization (EUA). The qRT-PCR reactions were prepared following the 200 μL sample input 96-well reaction plate according to manufacturer instructions, processed using an Applied Biosystems 7500 Fast Dx Real-Time PCR Instrument, and analyzed with the Applied Biosystems COVID-19 Interpretive Software (v1.5). The three SARS-CoV-2 viral genes recognized through this protocol were ORF1ab gene (ORF1ab), gene for the N protein (N), and gene for the S protein (S). The Ct values for the viral gene targets (ORF1ab, N, and S) had to be less than or equal to 37 for the samples to be identified as positive. Samples not meeting these criteria were excluded from further studies. The limit of detection (LoD) of SARS-CoV-2 viral concentrations was established within the TaqPath COVID-19 Combo Kit. 

### 2.3. NGS and Analysis

SARS-CoV-2 whole viral genome targeted NGS was performed by the UTEP-BBRC Biomolecule Analysis & Omics Core laboratory utilizing the following procedures. Invitrogen’s SuperScript IV First-Strand Synthesis System was used to produce cDNA from viral RNA. Next, IDT’s xGen for SARS-CoV-2 containing primers against the complete viral genome. Next, IDT’s xGen for SARS-CoV-2 containing primers against the complete genome SARS-CoV-2 isolate Wuhan-Hu-1 (NCBI Reference Sequence NC_045512.2) was utilized for library construction from first strand cDNA following the low viral load input plate protocol. Libraries were quantified with Qubit dsDNA high sensitivity kit and quality checked using a 4200 TapeStation with D1000 ScreenTape. Following quality check, libraries were normalized enzymatically with the IDT Normalase reagents. Equimolar library pools were sequenced in a MiSeq system from Illumina with V3 reagent kits of 600 cycles or using Illumina’s NextSeq2000 system with P2 (300 cycles) reagent kits while maintaining a consistent number of reads per sample. Because of the collective mutations arising in the SARS-CoV-2 genome and to achieve complete coverage in sequencing the genomes, it was necessary to expand the sequencing length from the 150 to 300 bases to allow for enough overlap of the sites with the increased number of mutations. Sequences of the samples were demultiplexed and saved in sample specific FASTQ files. Reads were trimmed for quality using Trimmomatic (v.0.38) and then aligned using Burrows–Wheeler aligner (bwa) to the Wuhan COVID-19 reference sequence (NCBI Reference Sequence: NC_045512.2) along with the GRCh37 human genome build as a decoy (v0.7.15). Sequences were then deduped and sorted using Samtools (v1.6). Bcftools (v1.12) *mpileup* was then used to create a compiled VCF file. Using Bcftools *consensus*, the sequence was extracted, and a single FASTA file of all the sequences was generated. Pangolin V (Aine, version 3.0), along with PUsher, was used to call the COVID-19 lineages from the generated FASTA sequences that had a maximum ambiguous cutoff of 20%. Generated results were combined, and graphs were plotted using pivot tables in Excel. Overall, 6.15% of samples sequenced were not assigned a lineage (224 unassigned) because of poor coverage and were excluded from further analysis and reporting in this study. Pango nomenclature was used to assign lineages that were reported as percent of the weekly total analyzed. 

### 2.4. Radial Phylogenetic Tree

To visualize the sequence relationship of the S-protein, FASTA sequences were loaded into Geneious Prime (v2022.2, www.geneious.com; accessed on 15 September 2022) and the S-gene was isolated. The S-gene was aligned using the Clustal Omega plugin (v1.2.3) and then translated. A Jukes-Cantor distance matrix was determined, and a Neighbor-Joining tree constructed utilizing the Wuhan S-protein was the outgroup. 

### 2.5. Data Availability

SARS-CoV-2 sequences were deposited to Global Initiative on Sharing All Influenza Data (GISAID) in accordance with data sharing requirements; sequences accessible by searching “TX-UTEP-” in the virus name field. Additionally, sequences are available at https://datarepo.bioinformatics.utep.edu/getdata?acc=YKQV0DBUXY130OL.

### 2.6. El Paso Strong Data

Data collected and reported by El Paso-DPH was obtained from the El Paso Strong website [17], accessed 8 January 2022. Data from year 2021 (wk 36 thru wk 52) to year 2022 (wk 01 thru wk 30) was plotted using Microsoft Excel to depict the number of new weekly cases, individuals hospitalized, and 7-day rolling average within the County of El Paso, Texas.

## 3. Results

### 3.1. COVID-19 Sample Characteristics

A collaboration between The University of Texas at El Paso Border Biomedical Research Center (UTEP-BBRC), University Medical Center of El Paso (UMC), and El Paso Department of Public Health (DPH) was formed to monitor SARS-CoV-2 variants within the southern U.S. border community through NGS. The study carried out by UTEP-BBRC sequenced 3641 SARS-CoV-2 positive samples provided by UMC and DPH, weekly, during a 48-week period from September 2021 to July 2022. An amount of 1635 samples were collected from UMC and 2006 samples from DPH. All samples were de-identified prior to analysis; thus, the study does not report gender, age, or symptomatic state. Table 1 indicates the number of samples sequenced each week. Sequenced samples remained steady (average 49 samples) during the initial phase of the study at the end of 2021 (wk 36-2021 to wk 49-2021). In January of 2022, an increase in samples were collected and examined (average 95 samples during wk 01-2022 to wk 06-2022). During wk 09-2022 to wk 16-2022, March 2022 and April 2022, fewer SARS-CoV-2 samples were received and sequenced. Finally, as the weeks progressed, we observed a gradual increase in samples reaching a maximum weekly count of 514 samples during July (wk 27-2022) and then tapering off towards the end of the study (wk 30-2022). 

### 3.2. SARS-CoV-2 Lineage Transition 

**Figure 1:**
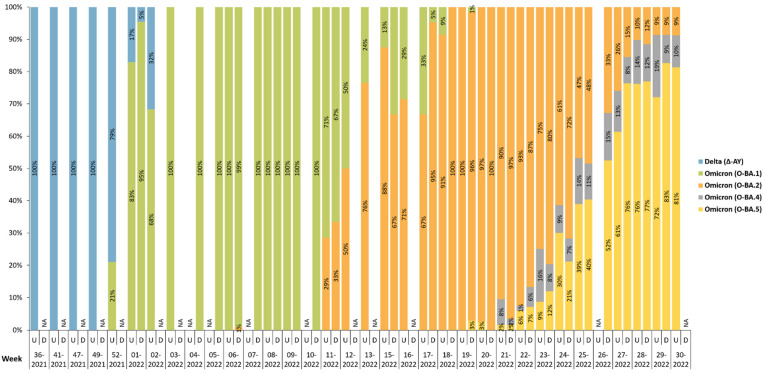
SARS-CoV-2 lineage distribution in the El Paso borderplex community. Major circulating variants and associated lineages were identified through sequencing of samples from UMC (U) hospitalized individuals or El Paso Department of Public Health (DPH) non-hospitalized community individuals. Lineages are illustrated as percent of total sequenced per week. Displayed are the percentage of samples identified as Delta (Δ-AY lineages) or Omicron (O-BA.1, O-BA.2, O-BA.4, and O-BA.5 lineages). NA, not available.

Variants were denoted according to the assigned WHO label, Delta (Δ) or Omicron (O). Greek letter symbols were used to denote these variants for easier visualization. The Pango lineage naming system was used to assign lineages and sublineages. Omicron variants were further characterized by the sublineages BA.2, BA.4, and BA.5. The predominant COVID-19 variants within this study were Delta and Omicron (Figure 1). Pango lineages that were identified in our study included: Δ-AY, O-BA.1, O-BA.2, O-BA.4, and O-BA.5. At the beginning of the study, the second week of September 2021 to the second week of December 2021 (wk 36-2021 to wk 49-2021), Delta was the only variant present within the sample population. The Omicron variant (BA.1 lineage; 21%) was detected during the last week of December 2021 (52-2021), which replaced the Delta variant. Lineage BA.2 appeared during March 2022, where it was observed in 29–33% of samples (wk 11-2022) during the third week and became the dominant strain by the final week (13-2022; 76% of samples). Two lineages, BA.4 and BA.5, appeared in May 2022 (wk 19-2022 to wk 21-2022). By June (25-2022), BA.4 and BA.5 were detected in 51–53% samples and became the major circulating lineages exceeding Delta and previous Omicron strains. Lineage BA.4 maintained its presence but failed to attain greater than 20% within our sample population. However, BA.5 proportions rapidly increased and was identifiable in up to 81% of samples during the final week of the study (30-2022).

### 3.3. Delta and Omicron Sublineage Spectrum

**Figure 2:**
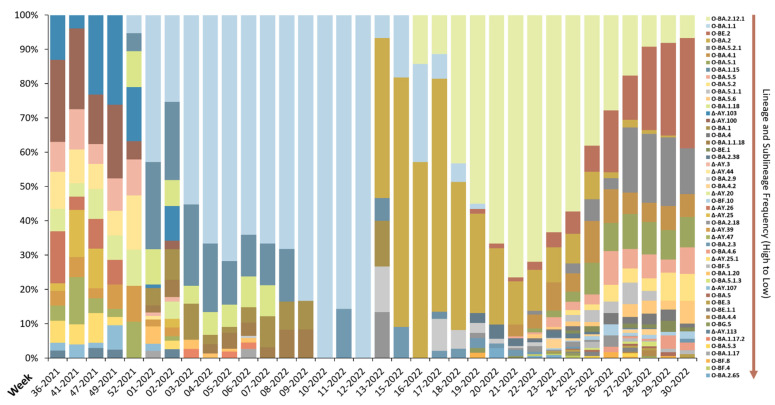
A diverse sublineage landscape of SARS-CoV-2 was observed in the evaluated samples from the El Paso community. Lineages and sublineages arising from the Delta and Omicron variants are illustrated as percent total weekly samples from the hospital and community, combined. A repertoire of 50 sublineages, are listed on the right in decreasing order of frequency within the study (high to low). Only lienages and sublineages identified in 5 samples or above are displayed. Delta-derived sublineages are denoted by Δ. Omicron-derived sublineages are denoted by O.

The appearance of SARS-CoV-2 lineages and sublineages from variants was evident as the original virus mutated. A total of 98 sublineages were identified in this study, 50 of which occurred in greater than 5 samples and are depicted in Figure 2. The most common sublineage was O-BA.2.12.1 (955 samples). The following sublineages emerged from the Delta lineage (24 sublineages, high to low frequency): AY.103, AY.100, AY.44, AY.3, AY.20, AY.25, AY.26, AY.47, AY.39, AY.25.1, AY.107, AY.113, AY.54, AY.75, B.1.617.2, AY.118, AY.43, AY.125, AY.39.1, AY.67, AY.117, AY.46.4, AY.16, and AY.74. The following sublineages arose from the Omicron BA.1 lineage (15 sublineages, high to low frequency): BA.1.1, BA.1.15, BA.1.18, BA.1, BA.1.1.18, BA.1.20, BA.1.17, BA.1.17.2, BA.1.21, BA.1.1.14, BA.1.15.1, BA.1.1.10, BA.1.1.16, BA.1.1.11, and BA.1.1.8. The following sublineages appeared from the Omicron BA.2 lineage (32 sublineages, high to low frequency): BA.2.12.1, BA.2, BA.2.38, BA.2.9, BA.2.18, BA.2.3, BG.5, BA.2.65, BA.2.7, BA.2.10, BA.1.1.2, BA.2.3.5, BA.2.3.14, BA.2.47, BA.2.37, BA.2.59, BA.2.1, BA.2.36, BA.2.20, BA.2.3.2, BA.2.23, BG.2, BA.2.13, BA.2.22, BA.2.3.17, BA.2.48, BA.2.50, BA.2.52, BA.2.56, BA.2.60, BA.2.75, and BA.2.9.3. The following sublineages originated from the Omicron BA.4 lineage (7 sublineages, high to low frequency): BA.4.1, BA.4, BA.4.2, BA.4.6, BA.4.4, BA.4.1.6, and BA.4.1.8. Finally, the following sublineages derived from the Omicron BA.5 lineage (20 sublineages, high to low frequency): BE.2, BA.5.2.1, BA.5.1, BA.5.5, BA.5.2, BA.5.1.1, BA.5.6, BE.1, BF.10, BF.5, BE.1.1, BE.3, BA.5, BA.5.1.3, BA.5.3, BF.4, BF.8, BA.5.1.6, BA.5.8, and BA.5.1.4. 

### 3.4. qRT-PCR S-gene Dropout 

**Figure 3:**
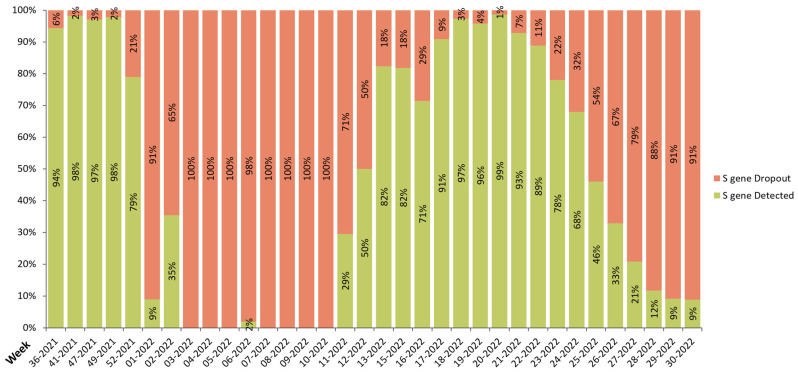
S gene detection trends observed in studied COVID-19 positive samples obtained from the border community. The TaqPath COVID-19 qRT-PCR assay was used to confirm the presence of SARS-CoV-2 in samples obtained during a 48-week period (week 36,-2021 to 30-2022) from both the UMC hospital and DPH general community. This assay detects three SARS-CoV-2 target genes, including the S gene for the spike (S) protein. The percent of total weekly samples that had S gene detection (green) or no S gene detection, i.e., S gene dropout, (orange) are depicted in the figure.

The failure to detect SARS-CoV-2 S-gene in COVID-19 molecular tests is referred to as S-gene dropout or drop-off [18]. The S-gene was detected in a vast number of samples during wk 36-2021 to wk 49-2021 (Figure 3). During the final week of 2021 (wk 52-2021), S-gene dropout occurred in 21% of samples and was widely present until the second week of March 2022 (wk 10-2022) (Figure 3). However, two weeks later (wk 11-2022), the appearance of the S-gene appeared again in 29% of samples (Figure 3). S-gene was detected at higher rates until the third week of May 2022 (wk 20-2022), where again a gradual increase of S-gene dropout occurred up until the end of the study (wk 30-2022) (Figure 3).

### 3.5. SARS-CoV-2 Viral Phylogenetic Tree

**Figure 4:**
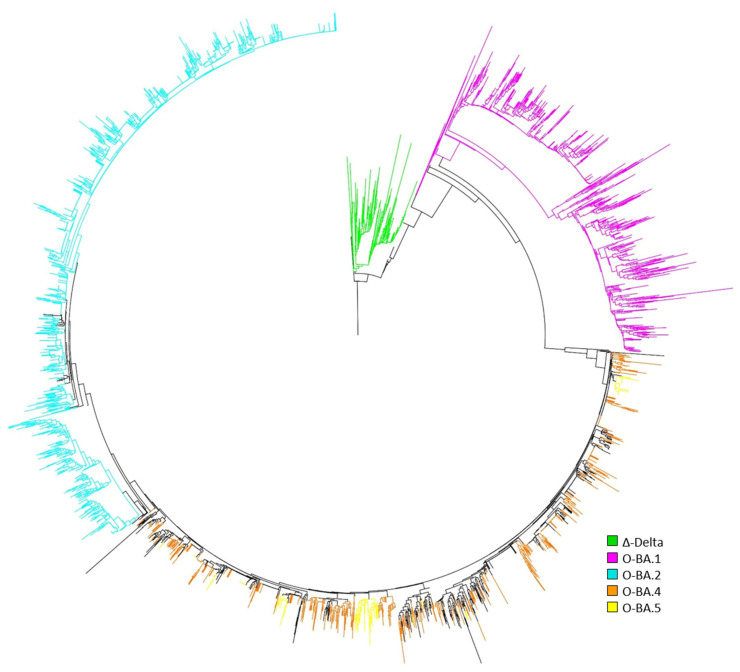
Neighbor-joining tree of S-protein alignment depicts evolutionary distance between Delta and Omicron samples collected during a 48-week period (wk 36-2021 to 30-2022). A radial phylogenetic tree was utilized to visualize the evolution of the S protein gene of SARS-CoV-2 in the studied sample population. Nodes of the tree were color coded with associated branches that contained only the same lineage among the nodes. Any branch that had more than one lineage associated with the leaves remained black. Green nodes represent the Δ-Delta variant. Other nodes represent lineages derived from the Omicron variant and are depicted as follows: magenta nodes for O-BA.1, blue nodes for O-BA.2, orange nodes for O-BA.4, and yellow nodes for O-BA.5. Height of tips indicate the amount of distance, relative to the reference, by individual samples due to mutations in the sequences.

The genetic distance between the SARS-CoV-2 S-gene within our sample population and the Wuhan S-gene as an outgroup was visualized through a radial phylogenetic tree (Figure 4). The Δ-strain (green within Figure 4) was observed to be tightly clustered while O-sublineages are spatially dispersed and indicate extensive genetic variance among those samples. BA.1 and BA.2 sublineages, magenta and blue respectively, formed monophyletic branches. BA.4 and BA.5, shown as orange and yellow, often had branches that were a mix. Within the phylogenetic tree (Figure 4), the height of the nodes suggest variability in the mutational load relative to the Wuhan S-gene reference. Here, Delta and O-BA.1 samples appear to have more variability in the S-protein, whereas O-BA.2, O-BA.4, and O-BA.5 possess fewer mutations overall.

### 3.6. El Paso Weekly Cases and Hospitalizations

**Figure 5:**
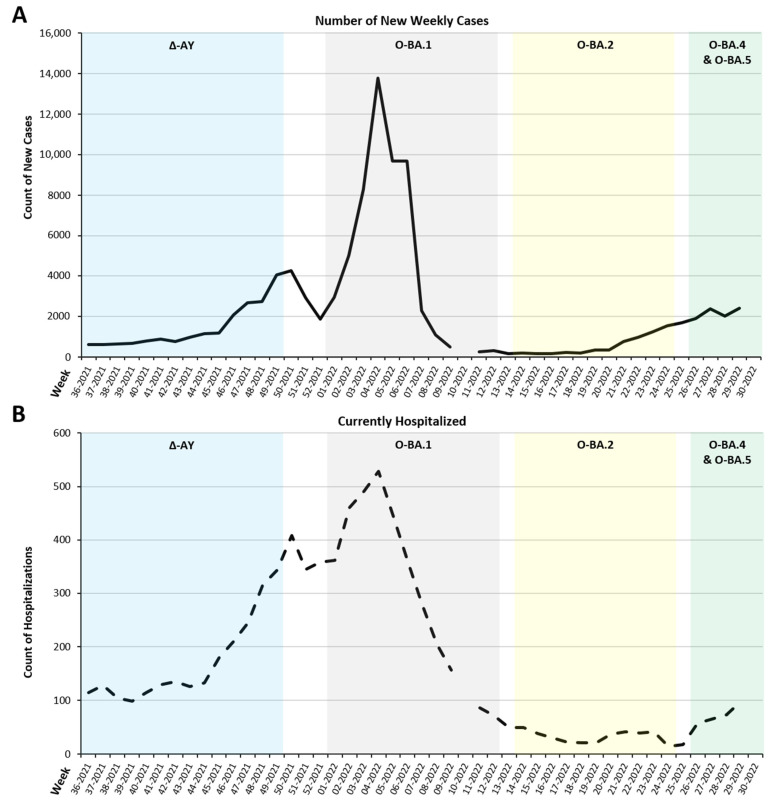
Trends in emergent COVID-19 cases and hospitalizations in the El Paso community. The City of El Paso reported new cases of COVID-19 infections and hospitalized COVID-19 patients on a weekly basis. Displayed are the counts, obtained from ElPaSOStrong (www.epstrong.org; accessed on 8 January 2022), of new cases and hospitalizations from September 2021 (week 36-2021) to July 2022 (week 30-2022), the same time period as the present study. (**A**) Weekly average of new cases of COVID-19. (**B**) Number of hospitalized patients due to COVID-19 infection. Breaks within the line graph for week 10-2022 and week 30-2022 represent unavailable data on ElPaSOStrong. The period when lineages found in the current study were dominant was represented by shaded boxes and superimposed to the graphs to visualize relationships between case or hospitalization counts and lineage appearance. Lineages are identified by the following: Δ-AY lineages by a blue box, O-BA.1 lineages by a gray box, O-BA.2 by a yellow box, and O-BA.4/O-BA.5 by a green box.

The incidence of weekly COVID-19 cases and hospitalizations within the El Paso community are displayed in Figure 5A,B. The number of cases remained steady until the second week of November 2021 (wk 45-2021) when the number of cases doubled (1170 vs 2691; 2.3 times). Then, cases dropped the last week of December 2021 (52-2021). An increase in incidence at the beginning of 2022 spanned four weeks, and it ended in a large peak of cases (13,786 cases) during the last week of January 2022 (wk 04-2022) and coincided with the presence of O-BA.1. COVID-19 cases decreased in the following weeks until they began to increase again around mid-May 2022 (wk 20-2022) when O-BA.2 was detectable. Hospitalization rates were similar to the trend of new cases, although on a smaller scale (Figure 5B). The largest peak in the number of hospitalized patients (529 patients) was observed during the same week that the largest number of new cases was observed (wk 04-2022). 

### 3.7. COVID-19 Cases in El Paso Community and in U.S. 

**Figure 6:**
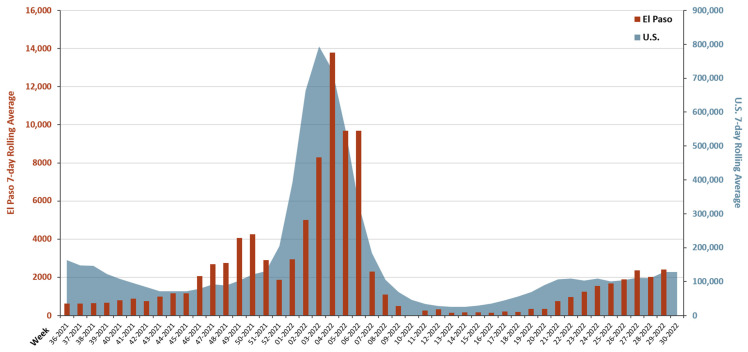
Trendline representation of COVID-19 cases in El Paso, TX and the United States (U.S.). Rolling 7-day average of new COVID-19 cases during the time span of September 2021 (week 36-2021) to July 2022 (week 30-2022) in El Paso (red bars) and the U.S. (blue shaded area) are depicted in the graph. The left y-axis (red) represents the scale for cases in El Paso. The right y-axis (blue) represents the scale for cases in the U.S. Breaks in the El Paso graph represent unavailable data. Data downloaded from ElPaSOStrong (www.epstrong.org; accessed on 8 January 2022) and CDC and Prevention COVID Data Tracker (https://covid.cdc.gov/covid-data-tracker/#trends_dailycases_select_00; accessed on 8 January 2022).

The trends in 7-day average national U.S. cases and El Paso community cases were similar except for an early peak that appeared in mid-November 2021 up to mid-December 2021 (wk 46-2021 to wk 50-2021) in new El Paso cases (Figure 6). 

## 4. Discussion

In this study, the viral genomic landscape of SARS-CoV-2 within the El Paso, Texas, border community was investigated. The observations illustrate the chronological progression of the Omicron variant within a predominantly Delta variant population of 3641 samples. Of these, 1635 samples were provided by UMC from hospitalized patients (Table 1). As the sole Level-1 Trauma Center in any 200-mile direction, UMC serves the El Paso, TX, USA-Juarez, Chihuahua, Mexico communities. Inpatient samples may have originated from symptomatic or asymptomatic individuals with the disease; however, patient information was dissociated from the specimens and not traced. In contrast, 2006 samples were provided by DPH, which were obtained from a COVID-19 testing location open to the community (Table 1). Throughout this study, samples were sequenced from the two locations. COVID-19 samples received were validated by qRT-PCR to determine a Ct value for at least two viral genes to confirm a continued SARS-CoV-2 positive status following their potential freeze/thaw and time from collection. Therefore, those that did not meet this criterion suggest low viral RNA presence for NGS and were not sequenced for further analysis. From UMC, we received 1969 samples, and 302 did not meet the study criteria for NGS. From DPH, we received 2408 samples, and 400 did not meet study criteria. Reliable sequence data of ≥80% genome coverage were obtained for approximately 90% of all samples received. We did not observe differences in the Pango lineage classification of variants between samples obtained from UMC and DPH (Figure 1). The inclusion of samples from different sources reduced bias and resulted in a more diverse sample cohort. 

The Delta and Omicron variants appeared successively as VOC in the U.S; as documented here, the evolution of SARS-CoV-2 variants were identified within our sample population from September 2021 to July 2022, (Figure 1). At the onset of this study, SARS-CoV-2 RNA was predominately found to be the Delta variant, primarily Δ-AY lineages (Figure 1 and Figure 2), from September 2021 (wk 36-2021) to December 2021 (wk 49-2021). Samples analyzed during this time represented 24 total sublineages of the Delta variant (Figure 2). The most common sublineage of the Delta variant was Δ-AY.103 (Figure 2), which first appeared in the U.S. in May 2020 [19]. Following the global arrival of Omicron (O-B.1.1.529), November 2021, the highly transmissible variant quickly replaced Delta (Δ-B.1.617.2) within the El Paso study population. Omicron first appeared during the last week of December 2021 (wk 52-2021), where an average of 21% of the samples were identified as the O-BA.1 lineage (Figure 1). By the third week of 2022, Omicron dominated Delta (Figure 1). Delta samples dropped from 32% to undetectable and all samples were identified as O-BA.1 (Figure 1). A significant reduction of COVID-19 cases within the U.S. attributed to Delta variant, similar to what is reported within this study and time period, has been sustained. Consequently, on April 14th, 2022, Delta was reclassified and no longer considered a VOC by the U.S. government SARS-CoV-2 Interagency Group (SIG). As time progressed, other lineages and sublineages arising from Omicron appeared. A total of 74 sublineages appeared for the Omicron variant (Figure 2). The most common sublineage for Omicron was O-BA.2.12.1 (Figure 2), derived from the O-BA.2 lineage, which originated in the U.S. and Canada in September 2021 [19]. With Omicron first observed in our data during the final week of December 2021, at the time of lineage transition there was only two weeks of data collection (wk 49-2021 and wk 52-2021), but missing data from the two-week period (50-2021 to 51-2021), inhibited our ability to monitor the spread of Omicron versus Delta. Lack of samples from the community (DPH) during the transition from Delta to Omicron was due to low numbers of reported cases. As of November 2022, Omicron lineages remain classified as a VOC by the WHO, the CDC, and the European Center for Disease Prevention and Control [19,20,21].

Recent data suggests the Omicron variant contains more mutations compared to its predecessors [22]. Lineages arising from the Omicron variant have several properties that permit the dominant behavior [23] and the ability to evade neutralizing antibodies obtained from previous vaccinations or infections, with BA.2.12.2 having the most significant capability [24]. The onset of O-BA.1 during the last week of December 2021 was associated with a spike in the rolling 7-day average of new weekly cases and hospitalized individuals in El Paso (Figure 5A,B), similar to the rest of the U.S. (Figure 6). The appearance of the O-BA.2 variant during the third week of March 2022 was associated with decreased weekly new cases and COVID-19 hospitalized individuals within El Paso (Figure 5A,B). Decreased cases was not expected since published literature refers to O-BA.2 as more transmissible and infectious than O-BA.1 [25,26]; therefore, external factors must have contributed to this trend. Indeed, the state of Texas reported low numbers during this same period in 2022 [27]. O-BA.2 persisted as the dominant lineage for about 10 weeks; thereafter, O-BA.4 and O-BA.5 emerged in the community (Figure 2). O-BA.4 and O-BA.5 have the same S-protein sequences [28]. However, in our study, O-BA.5 was prevalent at higher proportions (up to 81% of samples) than O-BA.4, which represented an average of 10.4% of weekly samples (Figure 2). 

New mutations within viral genes impact the effectiveness of molecular diagnostic tests, such as qRT-PCR, to detect SARS-CoV-2 [29,30]. The S-gene target is not recognized by the ThermoFisher TaqPath qRT-PCR kit for SARS-CoV-2 detection in samples containing the S-gene deletion 69-70 [31]. These events are commonly known as S-gene dropout, S-gene target failure (SGTF), or S-gene drop-off. The Omicron variant contains over 30 mutations within the S-gene, including the above mentioned 69-70 deletion [32]. The O-BA.2 lineage does not have the 69-70 deletion [32], which correlates with the S protein being recognized by PCR tests as was the case with the Delta variant. In previous studies, samples identified as Omicron (with the exception of the BA.2 lineage and sublineages) were associated with S-gene dropout, similar to what was observed with those samples identified to be Alpha (B.1.1.7 VOC 202012/01) [18,31]. Samples collected and sequenced in this study were associated with the S-gene dropout mirroring the progression of Omicron detection within the population (Figure 1 and Figure 3). S-gene dropout was observed in an average of 3.3% of samples analyzed from September 2021 to the second week of December 2021 (wk 36-2021 to wk 49-2021) (Figure 3). During this time, samples sequenced were associated with the Delta variant that, unlike Omicron, lacks the 69-70 deletion in the S protein (Figure 1) [33]. The last week of December saw an increase in S-gene dropout (21%) that was associated with the appearance of the Omicron O-BA.1 lineage (Figure 1 and Figure 3). S-gene dropout was observed at a higher frequency during the first week of January 2022 (wk 01-2022; 91% of samples) around the same time the Omicron variant became dominant (lineage O-BA.1, average 89% of samples) (Figure 1 and Figure 3). Not surprisingly, cases reported by the City of El Paso showed an increase in the number of active cases, new weekly cases, hospitalized individuals, and newly reported deaths following the emergence of O-BA.1 [17]. The appearance and successive increase in samples displaying the O-BA.2 lineage during the last week of March 2022 (wk 13-2022) caused an opposite trend in S-gene detection since there was a decrease in S-gene dropout (18% of samples) (Figure 1 and Figure 3). The introduction of lineages O-BA.4 and O-BA.5 followed by their dominance in the third week of June (wk 25-2022) resulted in an increase in S-gene dropout (54%) (Figure 1 and Figure 3). Lineage O-BA.5 remained dominant until the end of our study (wk 30-2022), and correspondingly, S-gene dropout continued to increase (up to 91%) (Figure 1 and Figure 3). Tracking the emergence of new lineages can be facilitated by the chronological observation of S-gene dropout of one or more targets in PCR tests [34]. Our study proves that S-gene dropout accompanies the appearance of new lineages that can be detected once PCR probes are updated to encompass new mutations. The timely identification of this trend is crucial to avoid false negative results in tested individuals. These data suggest that S-gene dropout can be used by health officials as an indicator of new SARS-CoV-2 variants in the community. Multiple global agencies have reported that S-gene dropout can assist in identifying Omicron variants [35,36,37]. 

In this study, the resulting S-protein sequences stemming from the Delta, O-BA.1, and O-BA.2 are clearly distinct clades (Figure 4). O-BA.2 showed a greater distance from the original Wuhan reference, whereas the O-BA.4 and O-BA.5 were intermixed but closer in similarity to the Wuhan reference. As seen in rodents, the viral fitness of O-BA.5 was greater than O-BA.2 in competitive experiments [38], suggesting that the additional mutations cause the lower fitness leading to the O-BA.5 to become the dominant lineage in our study. The intermixing of O-BA.4 and O-BA.5 were likely due to mutations that were not used in predicting the lineage by Pangolin and should be explored further to determine if additional clusters could be determined.

In the El Paso border region, peaks in the numbers of new weekly COVID-19 cases and hospitalizations during O-BA.1 were not seen with subsequent Omicron variants (Figure 5). Trends in the 7-day Rolling Average within El Paso closely paralleled the nation (Figure 6), with the exception of a peak in El Paso cases between mid-November 2021 to mid-December 2021 (wk 46-2021 to wk 50-2021) that was not seen in the U.S. data (Figure 6). Conducting genomic surveillance along the U.S. border regions can serve as an indicator of what is occurring nationally and inform the type of measurements that are needed against SARS-CoV-2, highlighting the impact of this study.

## 5. Conclusions

Case number trends within the El Paso community and U.S. cases reported by the CDC suggests that surveillance within the dynamic border community could be used to inform public health officials nationwide. This study tracked the progression of SARS-CoV-2 variants by the NGS of 3641 samples originating from the southern border community, including the general population and hospitalized individuals. No difference was observed in the frequency of variants expressed between samples that originated from hospitalized or non-hospitalized individuals. Omicron quickly replaced Delta as the most prominent variant detectable from individuals in the El Paso/Juarez borderplex community. The rise of Omicron, specifically the O-BA.1 lineage, was associated with the marked appearance of S-gene dropout. O-BA.1, O-BA.4, and O-BA.5 samples were associated with the S-gene dropout while the S-gene of Delta and O-BA.2 were largely recognized during qRT-PCR analysis confirmation of SARS-CoV-2. The evolution of the SARS-CoV-2 lineages in the El Paso/Juarez region followed a similar trend as other parts of the United States. Similar studies could be used to predict community control and prevention measures such as immunization strategies, healthcare preparedness, and COVID-19 testing availability to reduce viral transmission. 

## Figures and Tables

**Table 1:** Positive COVID-19 samples sequenced per week.

Week of the Year	UMC (Hospitalized)	DPH (Community)	Week Total
36-2021	53	-	53
41-2021	57	-	57
47-2021	70	-	70
49-2021	45	-	45
52-2021	19	-	19
01-2022	35	66	101
02-2022	82	-	82
03-2022	39	-	39
04-2022	-	76	76
05-2022	-	113	113
06-2022	54	106	160
07-2022	-	33	33
08-2022	77	9	86
09-2022	5	8	13
10-2022	-	4	4
11-2022	14	3	17
12-2022	2	-	2
13-2022	17	-	17
15-2022	8	3	11
16-2022	7	-	7
17-2022	15	84	99
18-2022	23	14	37
19-2022	2	69	71
20-2022	39	35	74
21-2022	63	119	182
22-2022	67	113	180
23-2022	92	167	259
24-2022	70	279	349
25-2022	77	99	176
26-2022	-	61	61
27-2022	243	271	514
28-2022	176	182	358
29-2022	93	92	185
30-2022	91	-	91
Grand Total	1635	2006	3641

## Data Availability

SARS-CoV-2 sequences were deposited to Global Initiative on Shar-ing All Influenza Data (GISAID) in accordance with data sharing requirements; sequences accessible by searching “TX-UTEP-” in the virus name field. Additionally, sequences are available at https://datarepo.bioinformatics.utep.edu/getdata?acc=YKQV0DBUXY130OL.

## References

[1] The International Bridges Steering Committee. https://pdnuno.com/.

[2] SARS-CoV-2 Variant Classifications and Definitions. https://www.cdc.gov/coronavirus/2019-ncov/variants/variant-classifications.html.

[3] Monitoring Variant Proportions. https://covid.cdc.gov/covid-data-tracker/#variant-proportions.

[4] Tracking SARS-CoV-2 Variants. https://www.who.int/activities/tracking-SARS-CoV-2-variants.

[5] Zhou B., Thao T.T.N., Hoffmann D., Taddeo A., Ebert N., Labroussaa F., Pohlmann A., King J., Steiner S., Kelly J.N. (2021). SARS-CoV-2 spike D614G change enhances replication and transmission. Nature.

[6] Hou Y.J., Chiba S., Halfmann P., Ehre C., Kuroda M., Dinnon K.H., Leist S.R., Schafer A., Nakajima N., Takahashi K. (2020). SARS-CoV-2 D614G variant exhibits efficient replication ex vivo and transmission in vivo. Science.

[7] Li Q., Wu J., Nie J., Zhang L., Hao H., Liu S., Zhao C., Zhang Q., Liu H., Nie L. (2020). The Impact of Mutations in SARS-CoV-2 Spike on Viral Infectivity and Antigenicity. Cell.

[8] Korber B., Fischer W.M., Gnanakaran S., Yoon H., Theiler J., Abfalterer W., Hengartner N., Giorgi E.E., Bhattacharya T., Foley B. (2020). Tracking Changes in SARS-CoV-2 Spike: Evidence that D614G Increases Infectivity of the COVID-19 Virus. Cell.

[9] Yurkovetskiy L., Wang X., Pascal K.E., Tomkins-Tinch C., Nyalile T.P., Wang Y., Baum A., Diehl W.E., Dauphin A., Carbone C. (2020). Structural and Functional Analysis of the D614G SARS-CoV-2 Spike Protein Variant. Cell.

[10] Rules for the Designation and Naming of Pango Lineages. https://www.pango.network/the-pango-nomenclature-system/statement-of-nomenclature-rules/.

[11] Variants, Sublineages, and Recombinants: The Constantly Changing Genome of SARS-CoV-2. https://www.rockefellerfoundation.org/case-study/variants-sublineages-and-recombinants-the-constantly-changing-genome-of-sars-cov-2/.

[12] Omicron Variant Accounts for 73 Percent of New COVID Cases in U.S. https://www.nbcnews.com/health/health-news/omicron-variant-accounts-73-percent-new-covid-cases-us-rcna9434.

[13] UT Southwestern Detects First Reported B.1.617.2 (Indian) Variant in North Texas. https://www.utsouthwestern.edu/newsroom/articles/year-2021/indian-variant-in-north-texas.html.

[14] Texas Reports Its First Case of the Omicron COVID-19 Variant. https://www.texastribune.org/2021/12/06/texas-omicron-covid-19/.

[15] Three Cases of Highly Infectious Delta Variant Confirmed in El Paso. https://kvia.com/coronavirus/2021/07/30/3-cases-of-delta-variant-of-covid-19-found-in-el-paso/.

[16] First 12 Cases of Omicron COVID-19 Variant Confirmed in El Paso. https://www.elpasotimes.com/story/news/health/2022/01/03/omicron-el-paso-covid-19-variant-first-cases-found/9086105002/.

[17] El PaSOstrong. https://www.epstrong.org/.

[18] Guerrero-Preston R., Rivera-Amill V., Caraballo K., Rodriguez-Torres S., Purcell-Wiltz A., Garcia A.A., Torres R.S., Zamuner F.T., Zanettini C., MacKay M.J. (2022). Precision health diagnostic and surveillance network uses S gene target failure (SGTF) combined with sequencing technologies to track emerging SARS-CoV-2 variants. Immun. Inflamm. Dis..

[19] Pango Lineages: Latest Epidemiological Lineages of SARS-CoV-2. https://cov-lineages.org/lineage_list.html.

[20] Summary of Variant Surveillance. https://covid.cdc.gov/covid-data-tracker/#variant-summary.

[21] SARS-CoV-2 Variants of Concern as of 10 November 2022. https://www.ecdc.europa.eu/en/covid-19/variants-concern.

[22] Aleem A., Akbar Samad A.B., Slenker A.K. (2022). Emerging Variants of SARS-CoV-2 And Novel Therapeutics against Coronavirus (COVID-19). StatPearls.

[23] Xia S., Wang L., Zhu Y., Lu L., Jiang S. (2022). Origin, virological features, immune evasion and intervention of SARS-CoV-2 Omicron sublineages. Signal Transduct. Target Ther..

[24] Cao Y., Yisimayi A., Jian F., Song W., Xiao T., Wang L., Du S., Wang J., Li Q., Chen X. (2022). BA.2.12.1, BA.4 and BA.5 escape antibodies elicited by Omicron infection. Nature.

[25] Lyngse F.P., Kirkeby C.T., Denwood M., Christiansen L.E., Molbak K., Moller C.H., Skov R.L., Krause T.G., Rasmussen M., Sieber R.N. (2022). Household transmission of SARS-CoV-2 Omicron variant of concern subvariants BA.1 and BA.2 in Denmark. Nat. Commun..

[26] Yamasoba D., Kimura I., Nasser H., Morioka Y., Nao N., Ito J., Uriu K., Tsuda M., Zahradnik J., Shirakawa K. (2022). Virological characteristics of the SARS-CoV-2 Omicron BA.2 spike. Cell.

[27] Tracking Coronavirus in Texas: Latest Map and Case Count. https://www.nytimes.com/interactive/2021/us/texas-covid-cases.html.

[28] Tuekprakhon A., Nutalai R., Dijokaite-Guraliuc A., Zhou D., Ginn H.M., Selvaraj M., Liu C., Mentzer A.J., Supasa P., Duyvesteyn H.M.E. (2022). Antibody escape of SARS-CoV-2 Omicron BA.4 and BA.5 from vaccine and BA.1 serum. Cell.

[29] Clark C., Schrecker J., Hardison M., Taitel M.S. (2022). Validation of reduced S-gene target performance and failure for rapid surveillance of SARS-CoV-2 variants. PLoS ONE.

[30] Soni A., Herbert C., Filippaios A., Broach J., Colubri A., Fahey N., Woods K., Nanavati J., Wright C., Orwig T. (2022). Comparison of Rapid Antigen Tests’ Performance Between Delta and Omicron Variants of SARS-CoV-2. Ann. Intern. Med..

[31] Bal A., Destras G., Gaymard A., Stefic K., Marlet J., Eymieux S., Regue H., Semanas Q., d’Aubarede C., Billaud G. (2021). Two-step strategy for the identification of SARS-CoV-2 variant of concern 202012/01 and other variants with spike deletion H69-V70, France, August to December 2020. Eurosurveillance.

[32] Zhou Y., Zhi H., Teng Y. (2023). The outbreak of SARS-CoV-2 Omicron lineages, immune escape, and vaccine effectivity. J. Med. Virol..

[33] Selected Characteristics of SARS-CoV-2 Variants of Concern. https://www.cdc.gov/coronavirus/2019-ncov/modules/variants/selected-characteristics-of-sars-cov-2-variants-of-concern.html.

[34] Bozidis P., Tsaousi E.T., Kostoulas C., Sakaloglou P., Gouni A., Koumpouli D., Sakkas H., Georgiou I., Gartzonika K. (2022). Unusual N Gene Dropout and Ct Value Shift in Commercial Multiplex PCR Assays Caused by Mutated SARS-CoV-2 Strain. Diagnostics.

[35] Threat Assessment Brief: Implications of the Emergence and Spread of the SARS-CoV-2 B.1.1. 529 Variant of Concern (Omicron) for the EU/EEA. https://www.ecdc.europa.eu/en/publications-data/threat-assessment-brief-emergence-sars-cov-2-variant-b.1.1.529.

[36] SARS-CoV-2 Viral Mutations: Impact on COVID-19 Tests. https://www.fda.gov/medical-devices/coronavirus-covid-19-and-medical-devices/sars-cov-2-viral-mutations-impact-covid-19-tests#:~:text=A%20specific%20deletion%20in%20the,fails%20to%20detect%20the%20virus.

[37] SARS-CoV-2 B.1.1.529 (Omicron) Variant—United States, 1–8 December 2021. https://www.cdc.gov/mmwr/volumes/70/wr/mm7050e1.htm?s_cid=mm7050e1_w.

[38] Uraki R., Halfmann P.J., Iida S., Yamayoshi S., Furusawa Y., Kiso M., Ito M., Iwatsuki-Horimoto K., Mine S., Kuroda M. (2022). Characterization of SARS-CoV-2 Omicron BA.4 and BA.5 isolates in rodents. Nature.

